# Replication-driven HBV cccDNA loss in chimeric mice with humanized livers

**DOI:** 10.1128/jvi.01295-25

**Published:** 2025-11-06

**Authors:** Bai-Hua Zhang, Yuanping Zhou, Stephen Horrigan, Fabien Zoulim, Jianming Hu, Yong-Yuan Zhang

**Affiliations:** 1 Virology, HBVtech 615170 , Rockville, Maryland, USA; 2 Department of Infectious Diseases, Nafang Hospital, Southern Medical University 70570 70570, Guangzhou, China; 3 Preclinical Department, Noble Life Sciences, Skysville, Maryland, USA; 4 UMR PaThLiv U1350, INSERM, Université Claude Bernard Lyon 1, Lyon Hepatology Institute, Hospices Civils de Lyon 26900 26900, Lyon, France; 5 Department of Microbiology and Immunology, Penn State College of Medicine 12310 12310 , Hershey, Pennsylvania, USA; Wake Forest University School of Medicine, Winston-Salem, North Carolina, USA

**Keywords:** persistent HBV infection, HBV replication, cytopathic effects, cccDNA clearance and replenishment

## Abstract

Hepatitis B virus (HBV) infection depends on the establishment of covalently closed circular DNA (cccDNA) and can be either transient or persistent. Persistent infection requires a noncytopathic viral phenotype, primarily achieved by limiting replication in infected cells. This study aimed to understand how most HBV-infected cells can avoid cytopathic consequences despite robust replication. Using chimeric mice with humanized livers, we assessed HBV replication kinetics and observed a progressive accumulation of viral products from early stages up to peak infection in infected cells, but the accumulation stopped increasing upon reaching the persistence phase, possibly resulting from the inhibition of HBV replication. We then examined HBV products, including cccDNA, transcription, and viral protein levels, to identify the potential point of inhibition, finding no clear suppression of viral transcription or protein synthesis. Quantitative analysis of cccDNA levels in bulk cells, single nuclei, and individual HBsAg-positive cells revealed that cccDNA was undetectable in a portion of infected cells. Our findings raise the possibility that infected cells may spontaneously clear cccDNA. This would stop HBV replication at its root and avoid the potential cytopathic consequences in infected cells due to uncontrolled viral replication. These results suggest that efficient cccDNA elimination might be achievable without directly targeting existing cccDNA.

**IMPORTANCE:**

The primary barrier to curing chronic HBV infection is the persistence of covalently closed circular DNA (cccDNA), which is traditionally considered stable within infected cells. However, clinical observations have revealed that cccDNA can undergo frequent clearance and replacement in patients with chronic HBV infection. Building on these observations, our study demonstrated that cccDNA was undetectable in a portion of HBV-infected cells at different timepoints after peak infection in chimeric mice with humanized livers, suggesting that spontaneous cccDNA clearance may occur. These findings align with clinical data and indicate that effective cccDNA elimination may be possible without the need to target cccDNA itself directly.

## INTRODUCTION

Hepatitis B virus (HBV) can cause persistent infection (1, 2). Establishing and maintaining HBV infection in hepatocytes requires the formation and persistence of episomal covalently closed circular DNA (cccDNA) molecules, which function as templates for viral transcription in the nucleus (3, 4). Therefore, a single copy of cccDNA in an infected cell is minimally required. The cccDNA molecules are assumed to be long-lived (5) because a chronic HBV (CHB) infection usually lasts for years or decades (6). CHB is thought to result from the failure of the host’s immune system to clear the established infection (7). Current HBV cure strategies aim to directly eliminate or permanently silence cccDNA (5) or kill infected cells in the liver (8).

Clinical evidence indicates that the cccDNA population in CHB undergoes dynamic evolution. For example, the wild-type (WT) viral population in serum or cccDNA in the liver can be cleared and replaced by mutant populations under both untreated and treated conditions (9–13). The HBe minus mutant replaced WT, then disappeared and reappeared again during an 18-month duration under untreated conditions (9). Or during a 36-month study involving lamivudine treatment, cessation, and retreatment, the dominant viral population shifted from WT to drug-resistant mutant, then to WT, and was subsequently replaced again by the drug-resistant mutant (14). These are examples that HBV cccDNA in CHB is subject to continuous clearance and replacement under different conditions.

Many viruses replicate efficiently within host cells that produce high intracellular viral load that must be processed, trafficked, and released (15). However, infected cells often cannot efficiently secrete virions and other viral products, further building up the intracellular viral load that causes stress in various cellular organelles, ballooning infected cells, and stretching and disrupting the cellular membrane, culminating in cell damage/destruction (16). This combination of robust replication and inefficient secretion may result in cytopathic or lytic infections, where the host cell is eventually destroyed (17, 18). Thus, a persistent viral infection in host cells is contingent upon noncytopathic effects of a virus, which are achieved mainly through controlling replication. For example, herpes simplex virus and human immunodeficiency virus (HIV) can establish persistent or latent infections through suppressing viral transcription after lytic or acute phase of infection (19–22).

Similarly, HBV replicates robustly, causing progressive retention of viral products in infected cells (23, 24). A continuous accumulation of viral products may also cause cytopathic effects. For instance, a common histopathology is ballooning degeneration in a portion of infected cells on liver sections (25), which resembles rounding of infected cells, a typical morphological manifestation of cytopathic effects observed in infected cultures (15). The retention of L protein in hepatocytes of HBV-transgenic mice causes a spectrum of pathologies, including hepatocyte enlargement and necrosis, and persistently elevated ALT levels. The severity of pathology is related to the concentration of intracellular envelope proteins (26). The intracellular HBsAg accumulation within smooth endoplasmic reticulum (ER) causes ER hyperplasia and displaces other organelles to the cell periphery, giving an appearance of “ground-glass” in some hepatocytes in CHB (27, 28). Cytopathic effects in infected primary hepatocytes and acute liver injury during the *in vivo* infection were also observed with an L protein mutant (G133E) of duck hepatitis B virus (DHBV), a member of *Hepadnaviridae* family known for congenitally establishing persistent but noncytopathic infections (1, 29). The observed cytopathic effects mainly resulted from increased intracellular levels of cccDNA, RNA, capsids, and rcDNA, as this L protein mutant impaired the production of enveloped virus (30, 31).

However, HBV infection is largely noncytopathic (32), suggesting that most infected cells can avoid cytopathic consequences. In theory, these infected cells must have the ability to suppress HBV replication following early robust replication, thereby preventing potentially cytopathic consequences. As observed in the DHBV model, DHBV replication is effectively brought under control at the late phase of infection at least through inhibiting recycling-mediated cccDNA replenishment (30, 33). This inhibition is mainly triggered by accumulating L protein levels in infected cells, which was later confirmed in HBV systems (34, 35). These studies demonstrate that (i) the cellular accumulation of viral products could trigger the inhibition of replication and (ii) the control of cccDNA levels is employed for controlling viral replication in infected cells.

In case of failure to control cccDNA levels, continuous cccDNA accumulation may cause cytopathic effects, leading to cell destruction (cccDNA loss) and cell regeneration for *de novo* infection (cccDNA replenishment), which could explain cccDNA loss in cells with cytopathic effects.

We are interested in understanding how HBV replication is restricted in most HBV-infected cells that avoid potentially cytopathic consequences using uPA/SCID chimeric mice with humanized livers. In this study, we analyzed cccDNA levels at both the single-nucleus and single HBsAg-positive cell level and the bulk cell level. Our findings indicate that cccDNA may be spontaneously cleared from infected cells in the humanized livers of chimeric mice, aligning with clinical observations of continuous cccDNA clearance and replacement (9–14). By confirming this clinical observation in our HBV model, we suggest that cccDNA elimination may be achievable by blocking cccDNA replenishment rather than directly targeting existing cccDNA.

## RESULTS

In this study, we characterized *in vivo* kinetics of HBV replication in uPA/SCID chimeric mice with humanized livers (36) and analyzed cccDNA levels using both bulk cells and single nuclei and HBsAg-positive cells.

**Fig 1:**
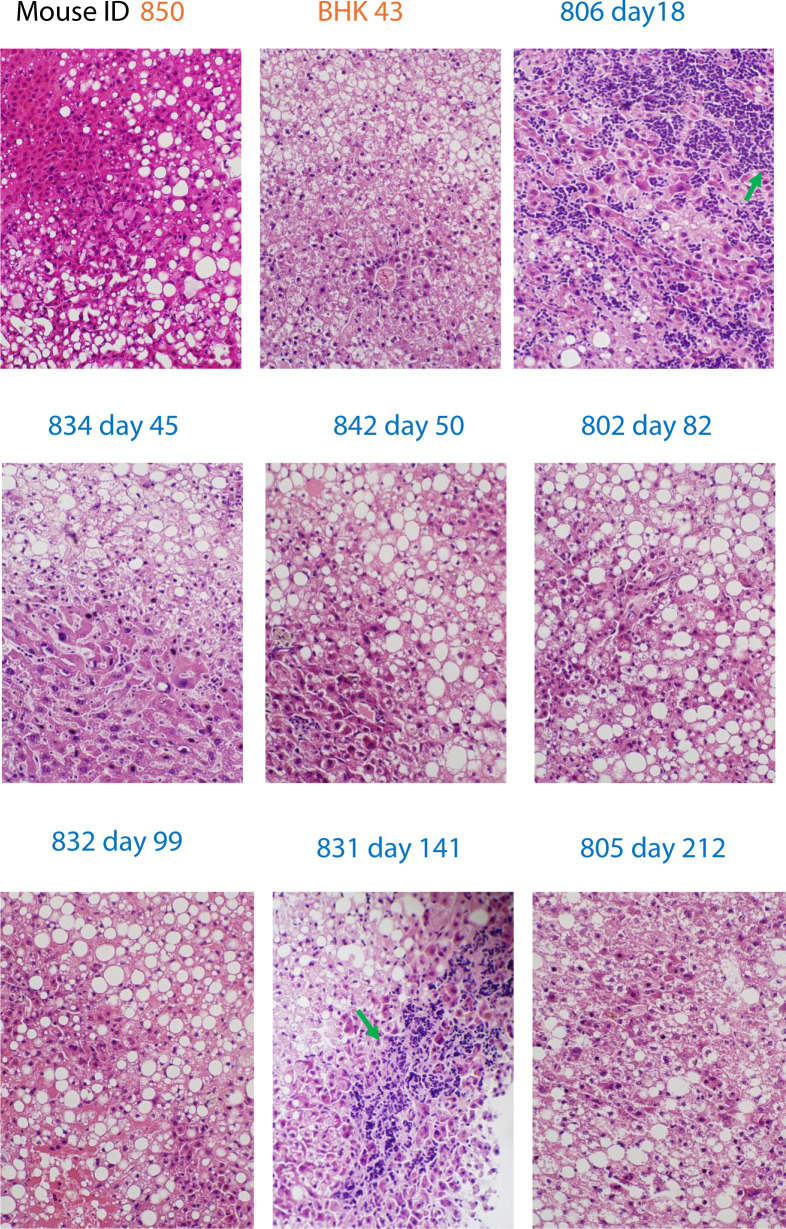
H&E staining sections (200×) of livers collected at different timepoint post-inoculation show mostly normal histology compared to uninfected liver sections. Mice ID 850 and BHK-43 in orange were uninfected. Mice ID 806, 834, 842, 802, 832, 831, and 805 in blue were infected and sacrificed on days 18, 45, 50, 82, 99, 141, and 212 pi. Focal areas with inflammation infiltrates are pointed with green arrows on sections 806 and 831.

This chimeric mouse model is known for supporting HBV persistent infection (37) that needs to be noncytopathic in most infected cells, though cytopathic effects involving a subset of cells were reported in this model (38). Similarly, we observed mostly normal histology of livers collected at various timepoints post-inoculation; however, focal areas of inflammatory infiltrates were present in some liver sections (Fig. 1). Therefore, the established persistent HBV infection, as well as dominantly normal liver histology in this model, supports that HBV infection in most cells is noncytopathic.

### *In vivo* replication kinetics suggested that the inhibition of HBV replication was possibly mediated through the clearance of cccDNA

**Fig 2:**
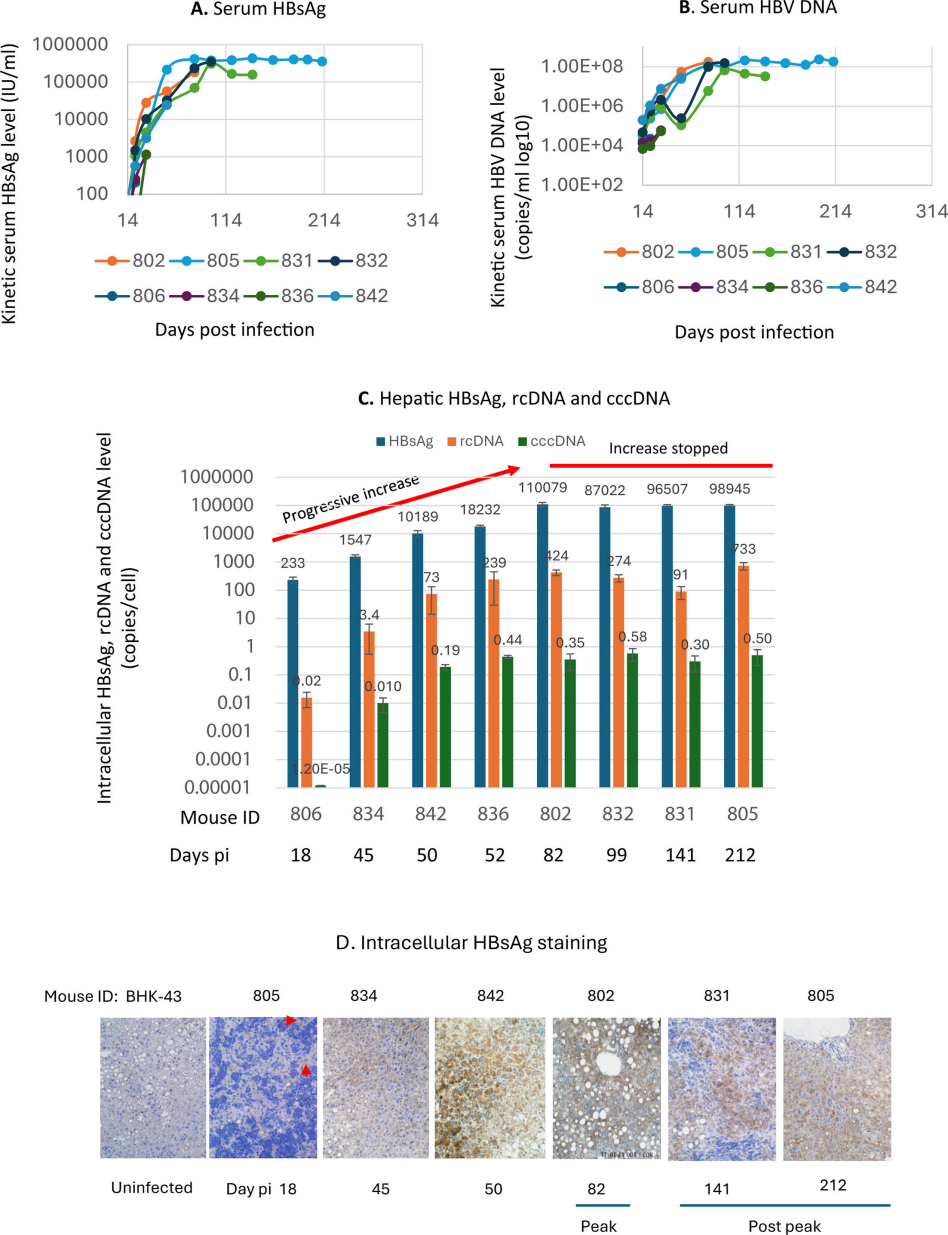
Intracellular accumulation of viral products is prevented from further increasing upon reaching the peak infection. (**A and B**) Kinetic serum HBsAg and HBV DNA levels. (**C**) Intrahepatic HBsAg, rcDNA, and cccDNA levels (copies/cell). Intracellular HBsAg was measured with ELISA. One mouse was sacrificed at each timepoint. (**D**) Immunostaining of intracellular HBsAg shows the spread of infection by increasing the number of HBsAg-positive cells, and it appears that most cells were HBsAg positive around day 50 pi. Red arrows indicate two HBsAg-positive cells on the day 18 section. pi: post-infection; cccDNA, covalently closed circular DNA; rcDNA, relaxed circular DNA. HBsAg, hepatitis B surface antigen. Error bars were plotted with standard deviations. Days pi indicates the day the mouse was sacrificed. 806, 834, etc. mouse ID.

To understand the kinetics of *in vivo* HBV replication, HBV-infected, untreated chimeric mice were euthanized on days 18, 45, 50, 52, 82, 99, 141, and 212 post-inoculation (pi) for assaying kinetic serum HBsAg and HBV DNA levels and intrahepatic HBV markers. There were two infection phases (Fig. 2A and B). The first was the phase of spread of infection to all infectible cells, in which both serum HBsAg and HBV DNA levels kept increasing after inoculation and peaked around day 82 pi. The second was the persistent infection phase, where HBV infection was maintained at a steady level. The observed kinetics of serum HBsAg and HBV DNA in this model recaptures the typical acute HBV infection that becomes persistent in humans (39).

The kinetics of intracellular accumulation of viral products also comprised two phases (Fig. 2C).

#### Phase of progressive increase in accumulation

The first was the phase of a continuous rise in the accumulation of viral products. For instance, intrahepatic HBsAg levels increased from 230 copies per cell on days 18 to 110,000 copies on day 82 pi, which reflects a robust HBV replication and that the secretion of virions and subviral particles lagged behind unrestricted HBV replication during the accumulation phase.

#### Phase of accumulation arrest

Following the peak on day 82 pi, the second phase began, where the increase in viral product accumulation stopped. During this phase, intracellular HBsAg levels remained steady around 100,000 copies per cell. Similarly, average cccDNA levels, as determined by qPCR using the standards calibrated with Absolute Q Digital PCR (ABQ dPCR), despite fluctuating twofold, stopped increasing over the next 130 days.

The steady levels of serum HBsAg and HBV DNA (Fig. 2A and B) suggest that the arrest in accumulation was not due to increased secretion of viral particles, assuming the cellular degradation of viral products and the clearance rates of viral particles from the blood remained stable, nor to significant cell death, which would have resulted in a reduction of both serum HBsAg and HBV DNA levels. Instead, the arrest likely resulted from an inhibition of replication, as accumulation would be expected to continue unabated if replication were not inhibited or to decline if the progressive accumulation persisted in most cells, potentially leading to significant injury to them. However, neither scenario was observed (Fig. 1 and 2), suggesting that replication may have been actively restrained to prevent cytopathic consequences in most infected cells. This two-phase pattern indicates a shift from active viral replication to a controlled state in late phase infection, essential for maintaining a persistent and noncytopathic infection in most cells (30, 33).

Our results also suggest that *in vivo* cccDNA kinetics includes two phases (Fig. 2C), and cccDNA may have been lost in both phases:

### Amplification phase

The total cccDNA level in the liver was amplified mainly by expanding the infection in the liver. The cccDNA level was increased from 0.00001 copies/cell (day 18 pi) to 0.35 copies/cell on day 82 pi (peak infection). Peaking infection means that all infectible cells must have been infected, as evidenced by the detection of HBsAg (Fig. 2D) in almost all hepatocytes upon the peak. The cccDNA level is expected to be ≥1 copy/cell because a minimum of one copy of cccDNA is required in each infected cell. However, the average 0.35 copies/cell at peak infection implies approximately 1 copy of cccDNA per 3 infected cells, suggesting that cccDNA after the initial establishment may have been lost in a portion of infected cells.

### Maintenance phase

cccDNA was maintained at a steady level (0.35–0.6 copies/cell) to sustain HBV infection at a steady level upon reaching the peak. With an average cccDNA level of <1 copy/cell, the Poisson distribution predicted that some cells may contain >1 copy/cell and other cells may contain no cccDNA. This suggests that cccDNA was possibly spontaneously cleared from some cells, which may involve noncytopathic clearance mechanisms in addition to possible cytopathic clearance in a subset of cells during the maintenance phase. Therefore, a steady HBV infection level in the persistent infection phase is reached, possibly by establishing an equilibrium between the number of infected cells with cccDNA that maintain HBV replication and those that have lost cccDNA and ceased viral replication.

### HBV RNA transcription was found to be highly efficient in infected livers

We explored whether HBV replication was subjected to inhibition. Three key stages of the HBV replication can be targeted to inhibit replication after peak infection: cccDNA, viral transcription, and viral protein synthesis. We first analyzed HBV RNA levels in infected livers.

The rcDNA extraction procedures retain cytoplasmic RNAs (40, 41) and were used for quantification of total HBV RNA levels by RT-qPCR.

Total HBV RNA levels were assayed in 80 rcDNA samples from four untreated livers (*n* = 20 per liver) to compare the relative RNA transcription efficiency between two phases of infection. Mice 842 and 836 were sacrificed on day 50 pi and 52 pi, respectively, representing the amplification phase. Mice 831 and 987 were sacrificed on day 141 pi and 218 pi, representing the maintenance phase.

**Fig 3:**
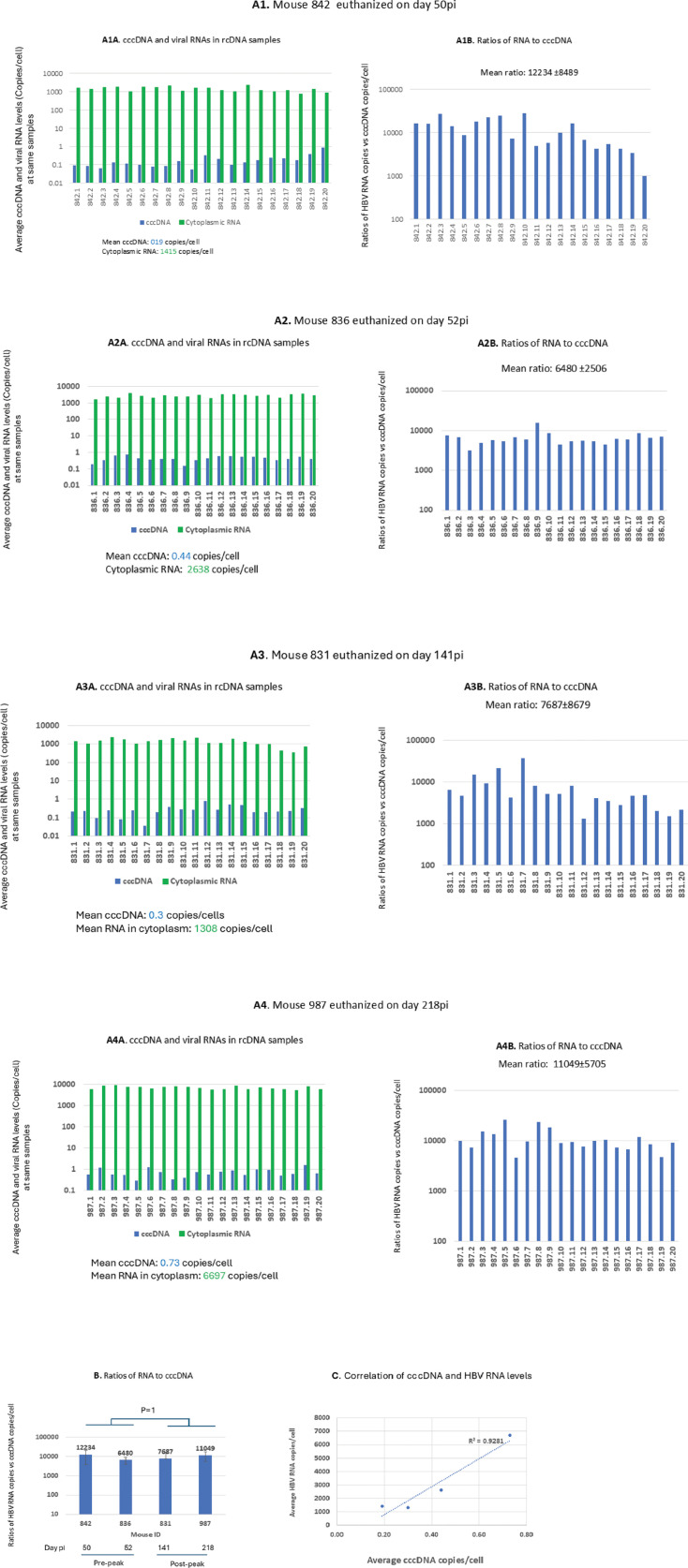
Average cytoplasmic HBV RNA levels among 80 liver samples. (**A1A, A2A, A3A, and A4A**) Average levels (copies/cell) of cccDNA and cytoplasmic HBV RNA were detected in mice 842, 836 (sacrificed at amplification phase), and 831 and 987 (sacrificed at maintenance phase). (**A1B, A2B, A3B, and A4B**) Ratios of HBV RNA copies/cell to cccDNA copies/cell in the same samples in mice of 842, 836, 831, and 987. (**B**) There were no significant differences in the ratios of average HBV RNA copies per cell to average cccDNA copies per cell before versus after peak infection. (**C**) Positive correlations between average cccDNA and total HBV RNA levels (copies/cell) among the same 4 mice. 842.1–842.20, 836.1–842.20, 831.1–831.20, and 987.1–987.20 represent sampling numbers for each liver.

Cytoplasmic HBV RNA levels exceeded 1,000 copies per cell in 74 of 80 samples (92.5%), ranging from 1,044 copies in sample 842.5 to 8,489 copies in sample 987.3. The remaining six samples had levels below 1,000 copies per cell, ranging from 351 copies in sample 831.19 to 981 copies in sample 842.13 (Fig. 3A1 through A4). The parallel kinetics observed in serum HBsAg and HBV DNA levels (Fig. 2A and B) during the two phases of HBV infection in this model suggest that HBV RNAs are predominantly transcribed from cccDNA. HBV RNA metabolism plays an important role in determining the steady-state levels of HBV RNA within infected cells. For example, pgRNA encapsidated within nucleocapsids is degraded during reverse transcription by the RNase H activity of HBV polymerase. In addition, HBV RNAs are also subject to degradation by host RNA decay pathways, and a fraction of pgRNA and HBx RNA may be packed into virions and secreted from cells (42). The detected HBV RNA reflects the remaining pool after metabolism. Despite these ongoing degradation and export processes, we observed that the intracellular HBV RNA levels remained relatively high given the low cccDNA levels, indicating that HBV RNAs are efficiently and continuously transcribed from cccDNA.

We used the ratio of RNA copies/cell to cccDNA copies/cell to measure the relative efficiency of RNA transcription from cccDNA. The high ratios of average RNA copies per cell to average cccDNA copies per cell in the same samples were notable (Fig. 3B1, B2, B3 and B4). Average ratios ranged from 6,480 to 12,232 in mice 836 and 842 representing the amplification phase and ranging from 7,687 in mouse 831 to 11,049 in mouse 987 representing the maintenance phase (Fig. 3B). These findings suggest that a single copy of cccDNA may undergo transcription up to 10,000 times, indicating efficient RNA transcription through repeated utilization of a single or few copies of cccDNA in HBV-infected cells. Notably, no discernible differences in relative transcription efficiency were observed between the two phases of infection (Fig. 3B). The observed steady HBV RNA levels may reflect ongoing *de novo* infection that replenishes HBV RNA in some cells that lost cccDNA, subsequently HBV RNA.

In addition, there was a positive correlation between average cccDNA and HBV RNA levels (R^2^ = 0.93, Fig. 3C), suggesting that total HBV RNA level is largely determined by cccDNA level in the infected cells of this model. Unlike the latent phase of HIV infection, at which viral RNA transcription in reservoir cells is inhibited, the efficient RNA transcription suggests that the suppression of RNA transcription is an unlikely mechanism to stop HBV replication, at least in this chimeric mouse system.

### No significant reduction in cellular envelope protein levels after peak infection

We compared HBsAg levels at both the peak stage (day 82 pi) and the post-peak stage (after day 82 pi). No marked difference in HBsAg levels was observed between these phases (Fig. 2A, C, and D), suggesting that the inhibition of viral protein synthesis is unlikely to be utilized as a mechanism to suppress HBV replication in this model. This is consistent with maintaining an efficient RNA transcription during the same phases described above.

### Average cccDNA levels after peak infection were less than 1 copy/cell

**Fig 4:**
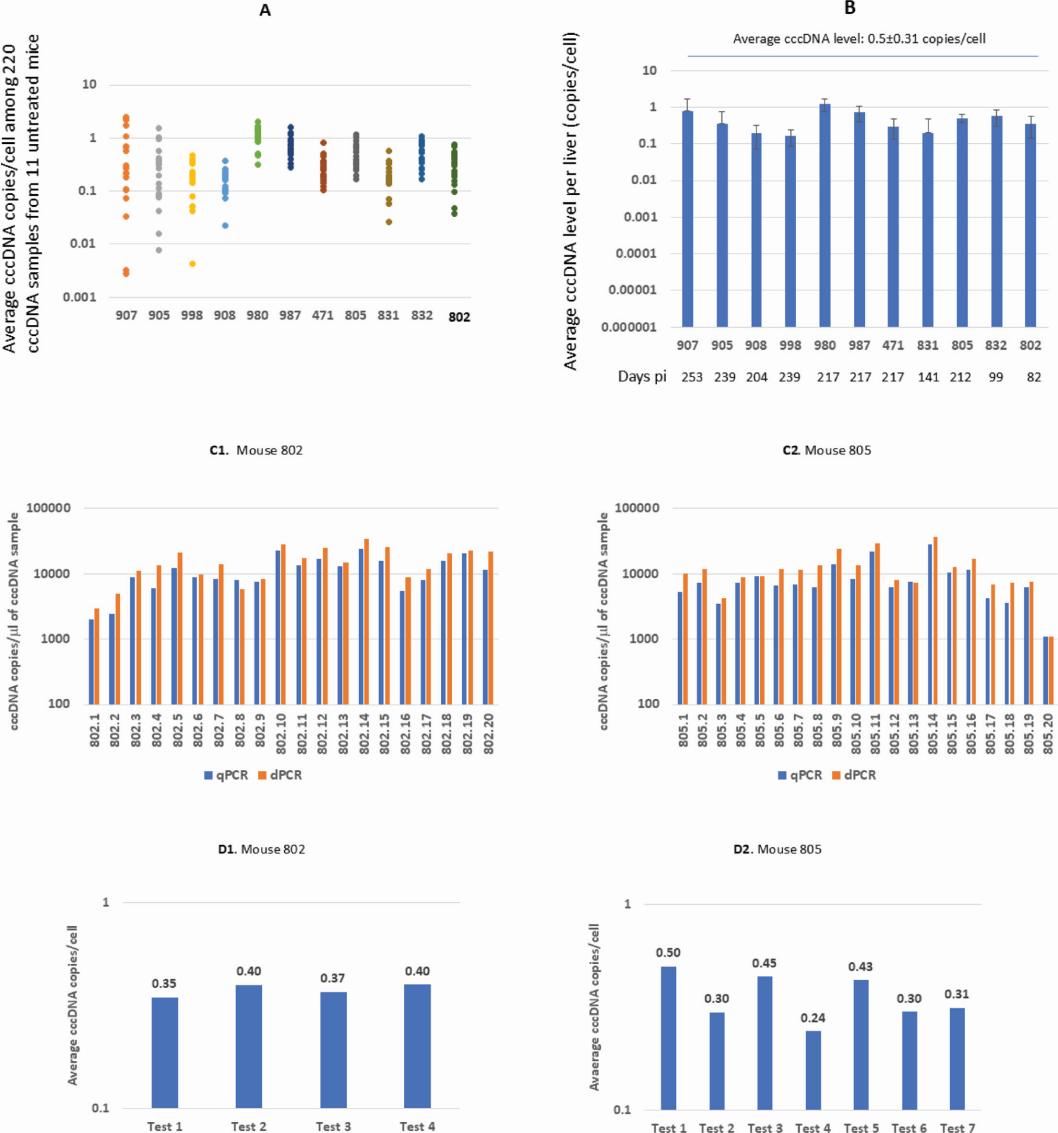
cccDNA levels in 192 of 220 cccDNA samples from 11 untreated mice were < 1 copy/cell. (**A**) Average cccDNA levels among 220 cccDNA samples. Each liver was randomly sampled 20 times, resulting in 20 cccDNA samples per liver. (**B**) Average cccDNA levels per liver among 11 untreated mice (blue). All 11 livers were collected after peak infection. Error bars were plotted with standard deviations. Days pi indicates the day the mouse was sacrificed. 907, 905, etc., are mouse IDs. (**C**) Comparison of cccDNA copies detected by both qPCR and ABQ dPCR in 20 cccDNA samples of mice 802 (**C1**) and 805 (**C2**). The detected cccDNA is expressed as copies/μL sample. (**D**) Evaluation of robustness of the cccDNA qPCR assay, showing the average cccDNA copies per cell for 4 and 7 independent tests of 20 cccDNA samples from mice 802 and 805, respectively. 802.1, 802.2, etc., or 805.1, 805.2, etc., indicate sampling numbers of that liver.

We extended the analysis of cccDNA levels in untreated mice to obtain the range of cccDNA levels in livers. To avoid non-representative findings by a single or a few samplings, we routinely sampled each liver 20 times, resulting in 220 cccDNA samples from 11 livers collected between days 82 and 253 pi. The highest average cccDNA level was 2.5 copies/cell, while the lowest was 0.003 copies/cell among the 220 cccDNA samples (Fig. 4A).

The average cccDNA levels in 28 (12.7%) of the 220 cccDNA samples were >1 copy/cell, whereas there were <1 copy/cell in the remaining 192 (87.3%) cccDNA samples, indicating that some cells may not contain cccDNA at different time points. The average cccDNA level in 220 cccDNA samples was 0.5 copies/cell.

The detected cccDNA level per liver varied considerably from 1.2 to 0.16 copies/cell among 11 livers. An average cccDNA level >1 copies/cell (1.2 copies/cell) was only detected in 1 of the 11 livers (Fig. 4B).

The ABQ dPCR method enables direct quantification of target molecules and serves as a reference standard for quantification. We compared cccDNA copies detected by qPCR and ABQ dPCR in 40 cccDNA samples from mice 802 and 805. As shown in Fig. 4C, the differences between the two assays are minimal, typically within a twofold range or less. Such variations are expected between two different assay platforms. This consistency indicates that the qPCR standards are well aligned with ABQ dPCR quantification, and the low cccDNA copies per cell detected are unlikely to result from underestimation by our qPCR assay.

We also evaluated the robustness of the cccDNA qPCR assay by retesting 20 cccDNA samples from mice 802 and 805 four and seven times, respectively. The results showed only fractional variations in mouse 802 samples, while up to twofold variations were exhibited in mouse 805 samples (Fig. 4D1 and D2), demonstrating the qPCR’s reliability across repeated measurements.

### cccDNA loss was detected at the single-nucleus level

One of the criteria used by the vendor PhoenixBio for selecting uPA/SCID chimeric mice with humanized livers is a liver replacement index (RI) of >70% (36). Among the 57 mice received, only four had RI between 76% and 78% and ranged between 80% and 93% in the remaining 53 mice, approximately 76% and 93% of liver cells being human liver cells. As bulk cells are routinely used for cccDNA quantification in our assay, we used an average of 30% non-human liver cells to normalize the calculated cccDNA copies/cell. The actual number of non-human liver cells varied in each sample, which may have impacted the calculated copies/cell. We sought to quantitatively detect cccDNA copies at the single-nucleus level (41) to corroborate the absence of cccDNA in some infected cells.

**Fig 5:**
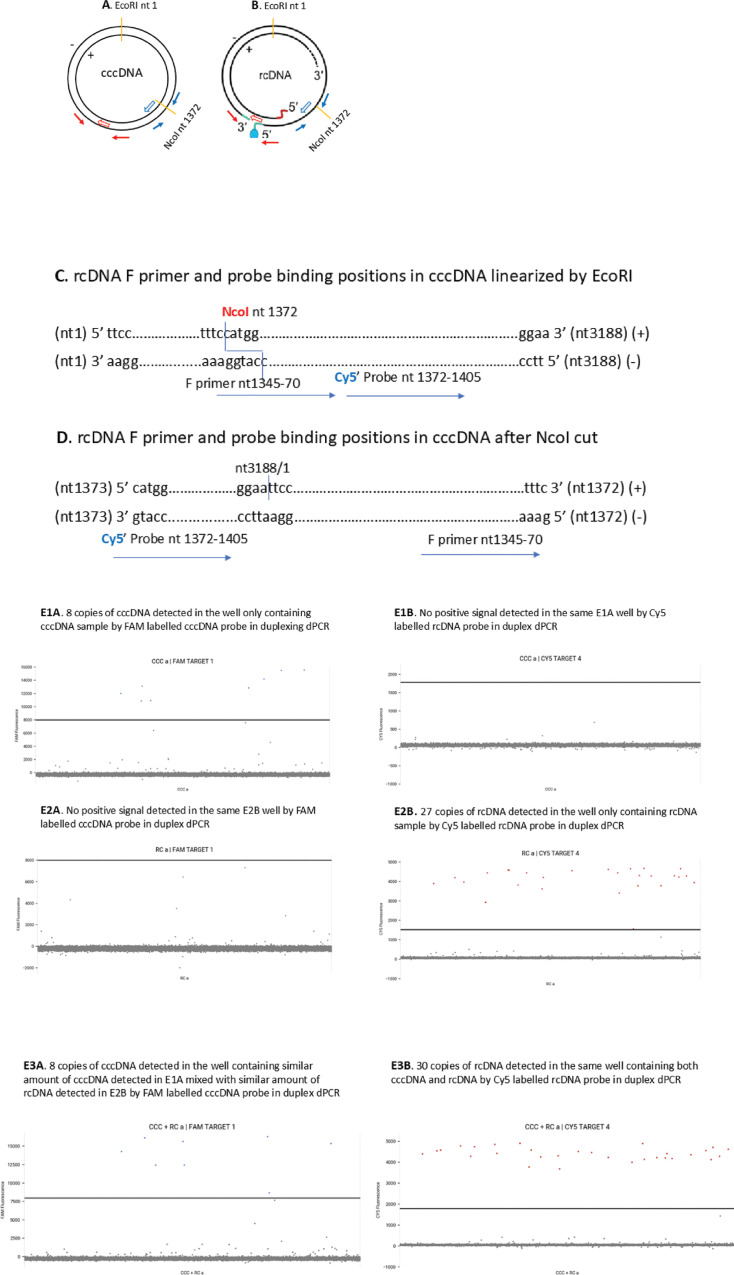
The NcoI linearized cccDNA template supports the specificity of rcDNA detection. (**A**) cccDNA is linearized with NcoI. (**B**) rcDNA cannot be cut with NcoI. (**C**) rcDNA F primer and probe binding positions in cccDNA template linearized by EcoRI. (**D**) Showing Cy5 dye labeled 1st 5′ base of the probe cannot be cut by Taq DNA polymerase on the NcoI cleaved cccDNA template. F primer. rcDNA forward primer. Blue arrows: rcDNA primers. Blue open arrow: Cy5-labeled rcDNA probe. Red arrows: cccDNA primers. Red open arrow: FAM-labeled cccDNA probe. cccDNA sequence (ADR v00867) is numbered from the EcoRI site. (**E**) Validation of the specificity of ccc and rcDNA detection by ABQ duplexing dPCR. Eight copies of cccDNA were detected in the well only containing cccDNA by FAM-labeled cccDNA probe in duplex dPCR containing both FAM-labeled cccDNA probe/primers and Cy5-labeled rcDNA probe/primers (**E1A**), but not by Cy5-labeled rcDNA probe in the same well (**E1B**). Twenty-seven copies of cDNA were detected in the wells only containing rcDNA by Cy5-labeled rcDNA probe (**E2B**) but not detected by FAM-labeled cccDNA probe by duplexing dPCR in the same well (**E2A**). Eight copies of cccDNA were detected in the well, containing a similar amount of cccDNA as detected in E1A mixed, with a similar amount of rcDNA as detected in E2B by FAM-labeled cccDNA probe in duplex dPCR (**E3A**). Thirty copies of rcDNA were detected in the same well by Cy5-labeled rcDNA probe in duplexing dPCR (**E3B**). The fractional differences in detected copies between rcDNA only and the rcDNA mixed with cccDNA likely resulted from variations in procedures, including pipetting samples.

HBV rcDNA is 100-fold to 1,000-fold more abundant than cccDNA and is also expected to be delivered to the nucleus for cccDNA conversion (43). We simultaneously detected cccDNA and rcDNA in each nucleus using Absolute Q duplexing digital PCR (ABQ duplexing dPCR), and the detected rcDNA was used as an HBV infection marker. The strategy, principle, and specificity of the simultaneous detection of cccDNA and rcDNA are described in detail in Materials and Methods and Fig. 5. However, this rcDNA detection may underestimate rcDNA copies if the rcDNA molecules in the nuclei were already undergoing a repair process that had filled the gap region of the plus strand, as they could be linearized by NcoI and excluded from the detection by duplex dPCR.

**Fig 6:**
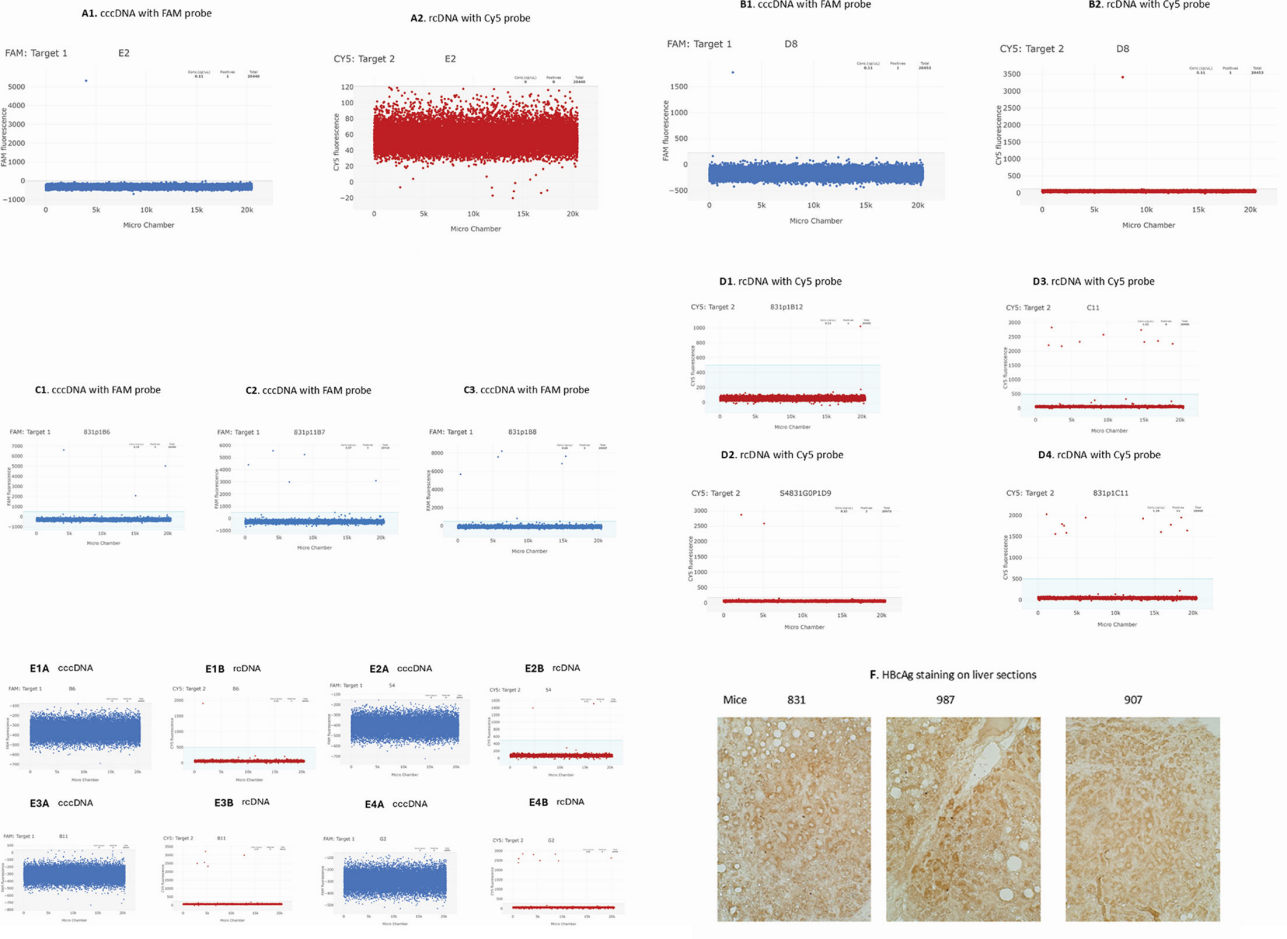
cccDNA and rcDNA detected at individual nuclei by ABQ duplexing dPCR. (**A**) A single copy of cccDNA was detected (**A1**), but no rcDNA was detected (**A2**) in the same nucleus. (**B**) A single copy of cccDNA (**A2**) and a single copy of rcDNA (**B2**) were detected in the same nucleus. (**C**) 3 (**C1**), 5 (**C2**), and 6 (**C3**) copies of cccDNA were detected in three different nuclei, respectively. (**D**) 1 (**D1**), 2 (**D2**), 9 (**D3**), and 11 (**D4**) copies of rcDNA were detected in four different nuclei, respectively. (**E**) cccDNA−/rcDNA+ nuclei. No cccDNA was detected in **E1A, E2A, E3A, and E4A**, while rcDNA was detected as 1, 2, 5, and 8 copies in corresponding nuclei (**E1B, E2B, E3B, and E4B**). (**F**) Immunohistochemical staining of HBcAg shows that most cells were core protein positive on sections of mice 831, 987, and 907.

Nuclei from three livers, harvested on day 141, 218, or 253 pi from untreated mice 831, 987, and 907, respectively (Fig. 6F), were deposited at one nucleus per well in a 96-well plate using a BD FACSAria II. The total number of analyses performed is listed in Table 1.

**TABLE 1:** Percentages of HBV-positive nuclei determined by duplexing dPCR

Mouse ID	No. of wells analyzed	No. of wells failed in dPCR	No. of successful wells in dPCR	No. of HBV-positive wells (%)
831	208	4	204	66 (32)
987	192	0	192	104 (54)
907	192	11	181	61 (33)

### Detected cccDNA and rcDNA at a single nucleus

cccDNA was detected as cccDNA only or coexisting with rcDNA (Fig. 6A and B), and rcDNA was detected coexisting with cccDNA or rcDNA only (Fig. 6B, E1B, E2B, E3B and E4B).

### cccDNA copies per nucleus

The cccDNA was detected as a single copy in most of the cccDNA-positive nuclei. Twenty (66.7%) of the 30 cccDNA-positive nuclei in mouse 831 contained only a single copy, while the remaining 10 nuclei had >1 copy, ranging from two to eight (Fig. 6C). In mouse 987, 41 (75%) of the 55 cccDNA-positive nuclei contained a single copy of cccDNA, while 14 (25%) nuclei had >1 copy. In mouse 907, the cccDNA was detected as a single copy in 34 (77%) of the 44 cccDNA-positive nuclei, while the remaining 10 nuclei contained >1 copy, ranging between 2 and 6 copies/nucleus. Thus, ≥2/3 of the detected cccDNA-positive nuclei contained only a single cccDNA copy.

### rcDNA copies per nucleus

A single copy of rcDNA was detected in 24 (57%) of the 42 rcDNA-positive nuclei in mouse 831, and the remaining 18 (43%) nuclei had 2–11 copies/nucleus (Fig. 6D). In mouse 987, a single copy of rcDNA was detected in 41 (60%) of the 66 rcDNA-positive nuclei, while the remaining 25 rcDNA-positive nuclei contained 2–8 copies/cell. In mouse 907, rcDNA was detected as a single copy in 13 (52%) of the 25 rcDNA-positive nuclei, and the remaining 12 (48%) nuclei had >1 copy of rcDNA, ranging from 2 to 19 copies/nucleus.

### cccDNA−/rcDNA+ nuclei

Despite the potential to underestimate rcDNA copies in the nuclei by our assay, a portion of infected cells from 27%, 47%, to 55% of the nuclei among the three livers (Table 2) had no detectable cccDNA, whereas rcDNA was detectable in the same nuclei (Fig. 6E). Ours (Fig. 2A and B) as well as published infection kinetics data (44) in this model show that peak infections are usually reached on days 82–90 pi, implying that all infectible human liver cells are likely already infected prior to days 90 pi. HBsAg and HBcAg staining showed that most cells were positive (Fig. 2D and 6F) in mice 831, 987, and 907 sections. Thus, cccDNA−/rcDNA+ cells likely represent either a loss of cccDNA from infected cells in which rcDNA was delivered through recycling or cccDNA is yet to be formed with the rcDNA delivered from *de novo* infection in recently generated uninfected cells after cccDNA loss.

**TABLE 2:** Percentages of cccDNA- and rcDNA-positive nuclei

Animal ID	Bulk cells	Positive percentage at single nucleus				
	cccDNA copies/cell	ccc+ only	ccc + rc	Total ccc+	rc+ only	Total rc+
831	0.3	36	9	45	55	64
987	0.7	37	16	53	47	64
907	0.8	59	14	73	27	41

The detected cccDNA−/rcDNA+ nuclei corroborated our bulk cell-based finding that cccDNA may have been spontaneously lost from a portion of the infected cells. The cccDNA loss observed during the maintenance phases of HBV infection, along with the presence of cccDNA−/rcDNA+ nuclei in three livers harvested after peak infection, suggests ongoing dynamic cccDNA turnover.

### Heterogeneous HBV-infected population demonstrated by FACS and ABQ duplexing dPCR-based analysis

**Fig 7:**
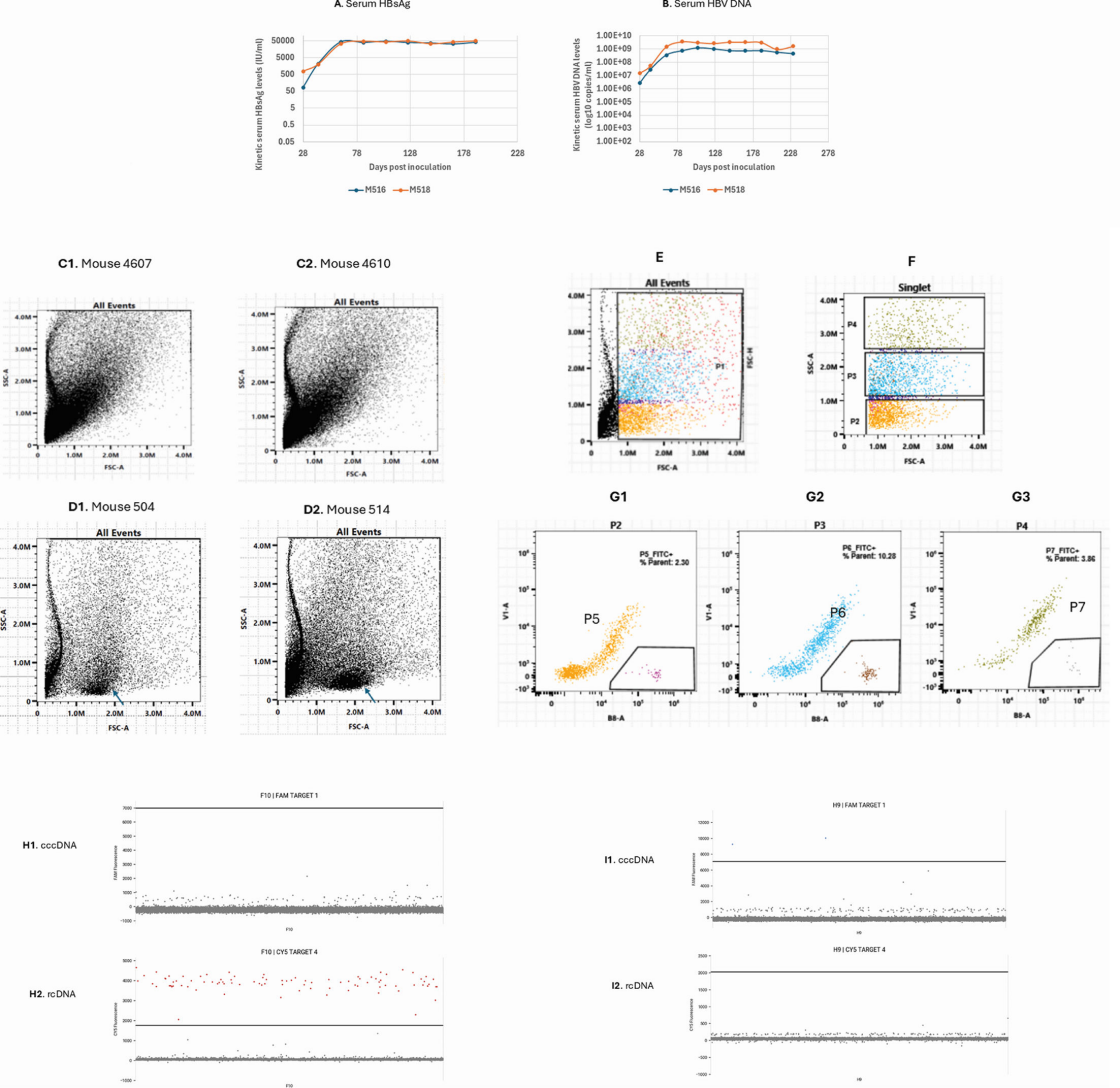
Heterogeneous HBV-infected populations detected by FACS and ABQ duplexing dPCR. (**A and B**) Kinetic serum HBsAg and HBV DNA levels in mice 516 and 518. (**C and D**) Illustrating cell populations using SSC-A and FSC-A measurements by Aurora CE Sorter. Distribution pattern of human liver cells isolated from two uninfected mice, 4607 and 4610 (**C**). Distribution pattern of human liver cells isolated from two infected mice, 504 and 514 (**D**). The arrow bar indicates a unique dense population of infected cells in each mouse. (**E**) Distribution pattern of human liver cells isolated from an infected mouse 518. (**F**) Singlets from mouse 518 were further gated into P2–P4 based on different SSC values. (**G**) FITC-positive cells from P2–P4 were further gated into P5–P7, respectively. (**H**) An example of HBsAg+/rcDNA+ (90 copies in red)/cccDNA- cell in H10 well. (**I**) An example of HBsAg+/rcDNA-/cccDNA+ (two copies in blue) cell at H9 well.

To further investigate cccDNA status in infected cells, we isolated HBsAg-positive cells through perfusion of the livers of two untreated HBV-infected chimeric mice (Mice ID 516 and 518) on day 228 pi. HBV infection reached the peak on day 84 pi and became persistent and maintained steady levels thereafter in these two mice (Fig. 7A and B). Isolated cells were stained with a rabbit anti-HBs antibody (LSBio LS-C683282), followed by a FITC-conjugated goat anti-rabbit IgG (LSBio LS-C60878) to gate FITC-positive cells for sorting.

We first analyzed the distribution pattern of isolated cell populations from two uninfected mice with humanized livers (Mice ID 4607 and 4610 provided by PhoenixBio) and two infected mice (ID 504 and 514) using standard side scatter (SSC-A) and forward scatter (FSC-A). In contrast to uninfected cells that are mainly distributed in the diagonally left region, the distribution of infected cells was spread into the diagonally right region, indicating an increase in both cellular granularity and size in a portion of infected cells. Notably, much larger sizes with twofold to threefold higher FSC values among the infected cells in the diagonally right region were detected in both infected mice compared to uninfected mice (Fig. 7C and D). Infected cells with different levels of virions, subviral particles, and capsids, factors that impact the extent of cellular granularity and size, are expected to have broader SSC and FSC values. There was also a major dense population with lower SSC but stretched along FSC to some extent (arrow in Fig. 7C and D). Therefore, a broader distribution of infected cell populations likely reflects a heterogeneous HBV-infected population with varying levels of viral components.

Such a wider distribution of infected cells was also detected in mice 516 and 518 (Fig. 7E showing mouse 518 FACS). Cells in Fig. 7E were further divided into three populations (P2–P4) based on increasing SSC values (Fig. 7F). HBsAg-positive cells within each of the P2–P4 populations were further gated based on FITC signals, resulting in P5–P7 (Fig. 7G1 through G3). Cells with higher SSC values indeed exhibited higher mean fluorescence intensity (MFI) (Table 3), indicating increased FITC intensity, that is, relatively higher HBsAg levels with the increased SSC values.

**TABLE 3:** Cells with higher SSC values are associated with cells with higher FITC intensities

Mouse ID	Mean FITC intensity^*a*^ of HBsAg-positive cells in three gated populations		
	P5	P6	P7
516	5.39E5	1.27E6	2.61E6
518	4.6E5	9.99E5	2.66E6

^*a*^ MFI values in the B8-A channel of Aurora CS Sorter were calculated by FlowJo v10.10.

To assess cccDNA status in HBsAg-positive cells from the P5 and P6 populations, gated HBsAg-positive cells were individually sorted into wells of 96-well plates. Due to the limited availability of cell suspension, P7 cells could not be collected. The DNA released from each well was then analyzed using ABQ duplexing dPCR to simultaneously detect both cccDNA and rcDNA within the same cells.

The cccDNA status in HBsAg-positive cells differed significantly between the P5 and P6 populations (Table 4).

**TABLE 4:** Duplexing detection of cccDNA and rcDNA in single HBsAg-positive cells^*a*^

	Cells with cccDNA only detected	Cells with rcDNA only detected	Cells with both cccDNA and rcDNA	Total
P5 (lower SSC)	28 (25%)^*a*^	74 (67%)^*a*^	8 (7%)	110
P6 (higher SSC)	68 (78%)^*a*^	13 (15%)^*a*^	7 (7%)	87

^*a*^ Chi-square value (X^2^) is 55.54 and *P*-value is 9.14 × 10^−14^ in comparison of the differences in the detected cccDNA only and rcDNA only containing cells between P5 and P6 populations.

In P5, most HBsAg-positive cells (67%) had detectable rcDNA but lacked detectable cccDNA (HBsAg+/rcDNA+/cccDNA−; Table 4; Fig. 7H). This pattern resembles the above-described nuclei that were rcDNA+/cccDNA−, supporting our finding that cccDNA was absent in a subset of infected cells. It also indicates that cccDNA could be the first viral DNA species cleared from these cells.

In P6, most HBsAg-positive cells (78%) contained detectable cccDNA but no detectable rcDNA (Table 4; Fig. 7I). This pattern may resemble the nuclei containing only cccDNA. These cccDNA-positive cells contained higher levels of HBsAg without detectable rcDNA, which could have been efficiently secreted as virions.

The difference in cccDNA-only and rcDNA-only cell populations between P5 and P6 was statistically significant (*P* = 9.14E-14; Table 4). The distinct patterns of HBsAg-positive cells observed in P5 and P6 provide additional support for our assay.

Combined results from FACS and duplexing dPCR revealed diverse states of viral components in infected cells isolated from the livers with persistent infection. They also provide new evidence that a subset of HBV-infected cells, which were positive for HBsAg and rcDNA, did not have detectable cccDNA. Given that cccDNA was absent in these freshly isolated HBsAg-positive cells, distinct from dead cells or debris per FACS gating, this finding suggests that the detected cccDNA loss in these cells was unlikely due to liver injury.

### Infrequent human Ki67 RNA expression indicated a low rate of human liver cell proliferation in humanized livers

Human Ki67 was chosen as a marker to examine the proliferation of human liver cells in our model and its potential association with observed cccDNA loss in the humanized livers of chimeric mice. This selection was made based on the previous staining of human Ki67 protein in the nuclei of human hepatocytes for proliferation studies within the same model (45). Ki67 RNA levels were measured using RT-qPCR. HepG2 cells and their derivative HepG2.2.15 cells are considered moderately proliferative, typically doubling in number every 2 days in culture. We used their cellular RNA as a control for Ki67 RNA expression, which averaged 32 copies per cell (*n* = 3).

We then analyzed Ki67 RNA levels in 380 cytoplasmic RNA samples derived from 19 humanized livers across four experiments: four livers collected on day 257 post-infection (pi) from experiment 1, three livers on day 218 pi from experiment 2, eight livers on days 18, 45, 50, 52, 82, 99, 141, and 212 pi from experiment 3, and four livers on day 230 pi from experiment 4.

**Fig 8:**
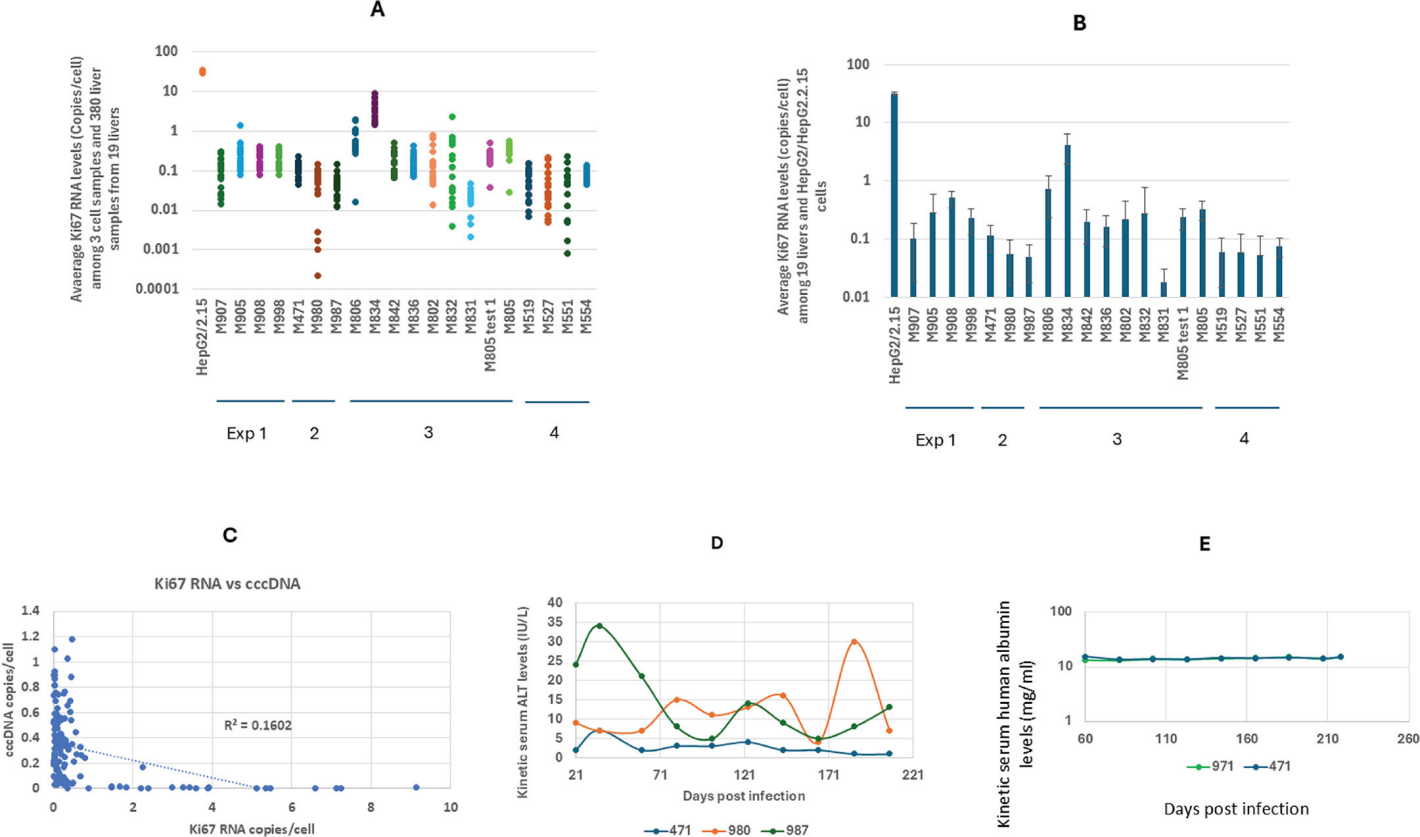
Average Ki67 RNA levels in liver samples and kinetic serum ALT and human albumin levels. (**A**) Average Ki67 RNA levels (copies/cell) among 380 rcDNA samples of 19 humanized livers collected from four different experiments indicated, as Exp1, 2, 3, and 4 under mice ID. Four livers were collected on day 253 pi for experiment 1, three livers collected on day 218 pi for experiment 2, eight livers collected on days 18, 45, 50, 52, 82, 99, 141, and 212 pi for experiment 3, and 4 livers collected on day 230 pi for experiment 4. HepG2/HepG2.2.15 cells were used as Ki67 RNA level control. Ki67 RNA levels in 20 samples of M805 were analyzed twice (labeled as M805 test 1 and M805, respectively), separated by approximately 16 months between the two tests. The results from the two tests were comparable. (**B**) Average Ki67 RNA levels per liver among 19 livers as well as from HepG2/HepG2.2.15 cells (*n* = 3). The same sample labeling system as in A. (**C**) Scatter plots of correlations of nuclear Ki67 RNA and cccDNA levels. (**D**) Kinetic serum ALT levels among three untreated mice. (**E**) Kinetic serum human albumin levels in two untreated mice. Error bars: standard deviations. M806, M834, etc., mouse ID.

Ki67 RNA levels were below one copy per cell in 355 of 380 samples (93%), and above one copy per cell in the remaining 25 samples (7%). Of these, 20 samples were from mouse 834, 4 from mouse 806, and 1 from mouse 905 (Fig. 8A).

At the liver level, the average Ki67 RNA was <1 copy per cell in 18 of the 19 livers, representing a 44 to 1,748 folds lower than HepG2/HepG2.2.15 cells (Fig. 8B). The remaining mouse, 834, exhibited an average of 4.1 copies per cell, still about eight-fold lower than HepG2/HepG2.2.15 cells.

These results indicate that Ki67 RNA expression was infrequent both across different time points during infection and among the four experimental cohorts.

If cell proliferation were a primary factor driving cccDNA loss, one would anticipate an inverse correlation between Ki67 RNA and cccDNA levels. However, no such correlations were detected between Ki67 RNA and cccDNA levels among 160 samples from 8 livers collected on days 18, 45, 50, 52, 82, 99, 14, and 212 pi, respectively (Fig. 8C).

### Normal serum alanine transaminase levels indicated the absence of significant liver injury during the infection course

Alanine transaminase (ALT) activity was assessed in serial serum samples from three untreated mice. ALT activity exhibited fluctuations over a 6-month duration, spanning from day 21 to day 207 pi, and remained below 40 U/L in all samples (Fig. 8D). This suggests that the observed reduction in cccDNA level occurred in the absence of significant liver injury.

We also monitored the potential loss of human liver cells in humanized livers by tracking kinetic serum human albumin levels, which remained steady (Fig. 8E). These findings are consistent with the observed low levels of human Ki67 RNA in the liver and normal serum ALT levels.

## DISCUSSION

We acknowledge that HBV cccDNA is conventionally viewed as stable in infected cells (5). Thus, cccDNA persistence in HBV infection can be simply explained by the longevity of cccDNA molecules. However, we also appreciate the extensive reports of clinical observations demonstrating the replacement of wild type with mutant population under treated or untreated conditions (9–13). Such turnover of viral population is also documented in the DHBV persistent infection model (46, 47). Furthermore, spontaneous cccDNA loss in DHBV-infected cultures was independently observed by two groups (48, 49). Thus, a dynamic cccDNA state is shared among members of the *Hepadnaviridae* family. Consistent with the clinically observed frequent cccDNA turnover (9–13), NA-treated human patients have demonstrated a 1–2.9 log reduction in cccDNA levels (50–55). Similar cccDNA reduction responses to NA therapy were also described in WHV-infected woodchucks (56), DHBV-infected ducks (41, 57), and HBV-infected uPA/SCID chimeric mice (45). Thus, the observed cccDNA reduction occurs across different species, constituting a broad biological base for this phenomenon. Since NAs do not directly target cccDNA molecules, these observations suggest that cccDNA may be spontaneously cleared from infected cells that did not experience cytopathic effects, in addition to a subset of cells that may have undergone cytopathic effects or spontaneous turnover that lost cccDNA through cell destruction or division.

Therefore, the notion of cccDNA longevity could not explain the dynamic state of cccDNA observed across several *hepadnavirus* species.

In this study, we detected spontaneous cccDNA loss through analysis of both bulk cells and single nuclei and single HBsAg-positive cells isolated from humanized livers of chimeric mice. Our findings confirm the observed cccDNA loss/reduction in human HBV infection (9–13) as well as in WHV (56) and DHBV infection models (41, 57) and in uPA/SCID chimeric mice model (45) under treated and untreated conditions.

**Fig 9:**
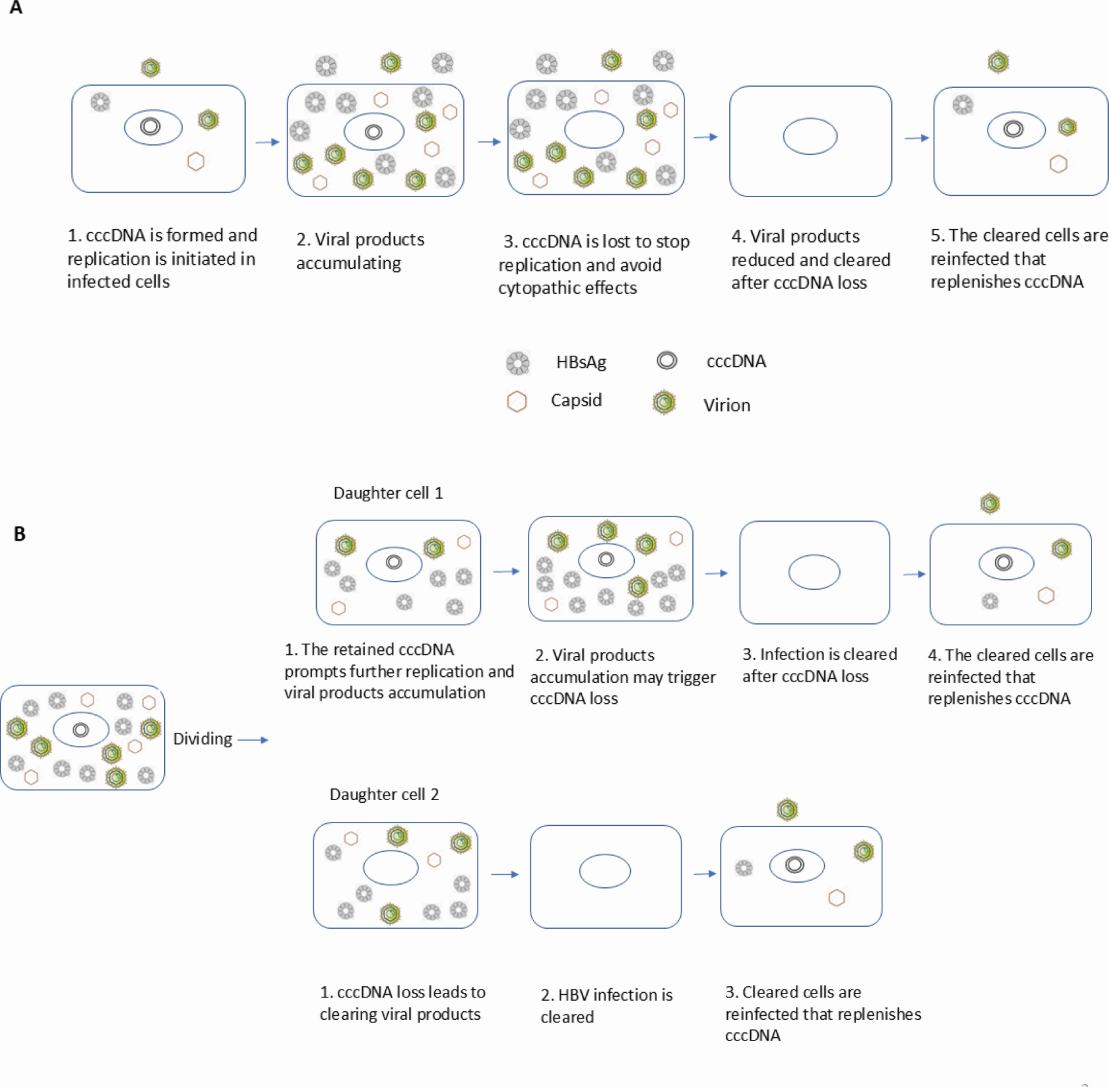
Proposed mechanisms for maintaining cccDNA persistence in infected livers. (**A**) Model illustrating how ongoing viral replication drives cccDNA loss in infected cells, leading to clearance of infection from individual cells. However, these cleared cells can be reinfected, resulting in cccDNA replenishment and sustained infection at the liver level. (**B**) Two proposed scenarios for cccDNA loss during hepatocyte division: Scenario 1: The parental cell divides, and daughter cell 1 retains the cccDNA. Continued replication in this cell eventually leads to cccDNA loss and infection clearance, but the cell can be reinfected to restore cccDNA. Scenario 2: Daughter cell 2 does not inherit cccDNA from the parental cell, leading to the loss of viral components and clearance of infection. This cleared cell is also susceptible to reinfection and cccDNA replenishment. These proposed mechanisms indicate that cccDNA persistence in the liver relies on continuous replenishment through reinfection, even as individual cells clear the virus through replication-associated cccDNA loss or cell division.

Our findings do not dispute an overall stable cccDNA level established after peak infection (Fig. 2C); rather, they provide an alternative explanation for how the persistence of cccDNA is maintained. Dynamic changes take place underneath the cccDNA persistence, that is, the early cccDNA pool is cleared either noncytopathically or through cell death/division, then the lost cccDNA pool is replenished. Therefore, the steady cccDNA level or cccDNA persistence is maintained by ongoing cccDNA replenishment after its loss (Fig. 9). We have also established preclinical proof that blocking cccDNA replenishment leads to progressive cccDNA elimination by >100-fold from infected livers (manuscript in preparation). Such therapeutic/functional evidence supports the notion that cccDNA persistence is mainly maintained by its ongoing replenishment, and the property of spontaneous cccDNA loss can be leveraged into a progressive cccDNA elimination.

Our findings appear to be able to explain both cccDNA persistence in CHB and clinically observed dynamic cccDNA clearance/replacement. They may also offer insights into why most infected hepatocytes avoid cytopathic consequences despite supporting early robust viral replication.

Liver injury certainly causes cccDNA loss, especially in untreated patients with CHB. However, the main scenario is different among the treated patients. For instance, the kinetics progressively replacing WT viral population were in parallel with progressively normalizing ALT kinetics, and the spread of the drug-resistant mutant occurred during a normal ALT period in four patients treated with lamivudine (14), suggesting that the observed replacements are probably independent of cell injury. This also seems to be held true for the observed 1–2.9 log cccDNA reduction (50–55) during NAs treatment that normalizes ALT levels in >80% treated patients, though spontaneous cell turnover or injury that led to cccDNA loss may still have occurred in a subset of cells of their livers.

Our findings, including largely normal histology, consistently normal ALT levels, infrequent Ki67 RNA expression, stable human albumin levels over infection course, and the presence of HBsAg-positive/rcDNA-positive cells lacking detectable cccDNA, suggest that spontaneous cccDNA loss occurs primarily through non-cytolytic mechanisms (58) in most cells that avoided cytopathic consequences, with a minor contribution from cytopathic clearance in a subset of cells with cytopathic effects during persistent HBV infection established in this chimeric mouse model.

We observed that the intracellular accumulation of viral products was likely halted at the late stage of infection (Fig. 2C). Our explanation is that HBV replication was probably inhibited. The analysis of levels of cccDNA, viral RNAs, and viral proteins indicates that the suggested inhibition may result from spontaneous cccDNA loss. This is why we assume the observed cccDNA loss is mainly driven by ongoing replication that causes progressive accumulation of viral products, which may successfully trigger the inhibition of replication at the cccDNA level, as reported in DHBV model (30, 33). In the case of unsuccessful inhibition of viral replication, cccDNA loss may also occur through cell injury in a subset of cells.

The limitation of this study is the lack of an estimate for the proportion of cccDNA loss attributable to cell injury and spontaneous cell turnover. Future studies are needed to further investigate the contribution of non-replication-related factors, such as natural hepatocyte turnover and proliferation to cccDNA decline in this model. A definitive measurement of cccDNA turnover *in vivo*, ideally at the single-cell level, is an important next step to generate additional evidence for cccDNA loss.

This study employed the Absolute Q digital PCR (ABQ dPCR) instrument to quantify cccDNA copies at single-nucleus and HBsAg-positive cell level. The ABQ dPCR is capable of detecting a single copy of the target gene. The dPCR assay is particularly suitable for detecting a low abundance of target molecules like cccDNA, as they can be individually distributed in a total of 20,480 microchambers. Each microchamber either contains zero or a single copy of cccDNA, reduces competition among templates, dilutes out inhibitory factors, and minimizes variability.

While we are confident in the single-copy detection sensitivity of ABQ dPCR, we cannot entirely rule out the possibility that some undetectable cccDNA in certain cells may reflect the limits of detection.

Early in our study, we carefully investigated all potential technical sources of variability, including reagent quality, extraction protocols, sample handling and storage conditions, and qPCR assay performance and standard calibration, and found no evidence of procedural inconsistencies that could account for the observed differences.

Importantly, our flow cytometry-based analysis of HBsAg-positive cells revealed two distinct subpopulations, suggesting that the absence of detectable cccDNA is not solely due to technical limitations.

Average cccDNA levels varied considerably among 11 livers. We believe the variability is more likely due to biological and virological factors, particularly heterogeneity in the transcriptional activity of individual infected hepatocytes. Specifically, the relative usage of HBV promoters (core vs. surface) may differ among cells, leading to divergent transcriptional profiles. As observed in human liver biopsies, some cells are core-positive only (indicating preferential pgRNA transcription, consequently higher rcDNA levels, and potentially more active rcDNA-to-cccDNA recycling or higher cccDNA levels), while other cells exhibit only HBsAg staining (likely reflecting dominant S RNA transcription and limited pgRNA expression, resulting in lower rcDNA and cccDNA levels) (59). The proportions of these transcriptionally distinct hepatocyte subpopulations may vary among animals, contributing to the liver-to-liver variability in average cccDNA levels.

Our FACS results revealed that a portion of infected liver cells was widely distributed in the diagonally right regions compared to an uninfected liver cells population, suggesting a heterogeneous infected cell population with varying levels of viral products. In addition, we observed that only about 7% of HBsAg-positive cells contained both cccDNA and rcDNA. This pattern is reproducible when sorting from the same regions with low SSC values from two additional infected humanized livers. We acknowledge, however, that cells from other regions were not gated and analyzed in this study, and the proportions may differ. Interestingly, this finding resembles immunohistochemical observations in liver sections from both HBeAg-positive and -negative patients, where fewer than 10% of infected hepatocytes showed dual positivity for HBsAg and HBc proteins, and only detected in HBeAg-positive patients (59). This supports the possibility that most infected cells are mutually exclusive in terms of expressing HBsAg and HBc, potentially reflecting mutually exclusive transcription of pgRNA versus S RNA. Such exclusiveness may partially explain why only a small proportion of HBsAg-positive cells harbor both cccDNA and rcDNA. These observations may also suggest that turnover of cccDNA and rcDNA in infected hepatocytes could be rapid, implying that persistent HBV infection could be interrupted and terminated if new rounds of infection are durably blocked, which worked efficiently when this new strategy was tested in our chimeric mouse model (manuscript in preparation). However, our analyses were preliminary and limited to establishing proof for the absence of detectable cccDNA in some HBsAg+/rcDNA+ cells. More detailed analysis is warranted in future studies.

Our findings prompt us to rethink the cccDNA elimination strategy. Directly targeting cccDNA or killing infected cells is currently thought to be the only pathway for cccDNA elimination and complete cure of chronic HBV infection. Our results provide an additional strategy that does not require direct targeting of cccDNA molecules and may achieve cccDNA elimination through durable blocking of cccDNA replenishment.

## MATERIALS AND METHODS

### Animals and HBV infection

uPA/SCID chimeric mice were supplied by PhoenixBio USA (New York, NY, USA). All mice were kept in housing cages (TP107, One Corporation, Osaka, Japan) in a BSL-2 room with controlled temperature at 23°C and 12 hour-light/dark cycle. All animals were fed with γ-radiated CRF1 food and autoclaved water *ad libitum*. An HBV inoculum, prepared from mouse serum (project no H01-108 animal 4) by diluting viremia of 5E9 HBV DNA copies/mL to 2E7 HBV DNA copies with PBS in 100 µL volume, was administered intravenously (tail vein) to each chimeric mouse.

### Monitoring HBV infection and human albumin level in blood

Blood was collected tri-weekly for quantification of serum HBV DNA (qPCR, see below), HBsAg (GS HBsAg EIA 32591, Bio-Rad), and human albumin (Human albumin ELISA kit E-80AL, Immunology Consultants Laboratory) levels by ELISA per instructions.

### ALT activity in serum

Serum ALT activity was assessed using the alanine transaminase colorimetric assay kit (Cayman Chemical item no 700260) according to the detection manual. Absorbance values were measured at 340 nm once per minute for 10 min, and the resulting 10 absorbance values were plotted against time. Due to limited serum volumes, a modification was made: the 20 µL serum sample was adjusted to 10 µL and compensated with 10 µL of H2O. Consequently, in the calculation formula, the 0.02 mL of serum sample was adjusted to 0.01 mL accordingly.

### Analysis of intrahepatic HBV DNA

Each liver was randomly sampled 20–40 times by cutting 20–40 mg liver tissue (weighed and recorded) and placed in a disposable micro-homogenizer (BioMasher, Takara cat no: 9790B) in 500 µL of an isotonic buffer (154 mM Tris-HCl, pH 7.5, 1 mM EDTA, and 0.05% Triton X-100) with 10 strokes. The homogenized tissue suspension was spun for 2 min at 14,000 rpm. The aqueous phase (about 400 µL) was transferred to a new microtube for isolation of replicative intermediates (RI) while the nucleic pellet remained in the tube for cccDNA isolation.

Note: Two negative controls were included for each round of extraction, one placed in the first sample position and the other in the last position to monitor any contamination during extraction.

### Extraction of rcDNA from the 400 µL supernatant by following procedures

Mix lysates with 110 µL of proteinase K (final concentration: 0.5 mg/mL) and 1% SDS, and incubate at 50°C for 1 h. Add 500 µL phenol, vortex, chill on ice for 3 min, and centrifuge at 14,000 rpm for 2 min. Transfer the supernatant to a new tube, add 1,000 µL of 100% ethanol for precipitation, and centrifuge at 14,000 rpm for 15 min. Wash the pellet with 1,000 µL of 100% ethanol, followed by centrifugation at 14,000 rpm for 10 min. Remove residual ethanol and air-dry the pellet for 5 min. Dissolve the pellet in 200 µL of 10:1 TE buffer (pH 7.4). The resulting rcDNA is ready for qPCR analysis.

### Extraction of cccDNA from nucleic pellets by following procedures

The nuclear pellet was suspended in 200 µL of 10:1 TE buffer containing 0.05% Triton X-100 (pH 7.4), followed by the addition of 200 µL of 6% SDS–0.1 M NaOH solution and incubation at 37°C for 15 min. Next, 100 µL of 3 M potassium acetate (pH 5.07) was added, mixed thoroughly, chilled on ice for 5 min, and centrifuged at 14,000 rpm for 2 min to remove KSDS–protein–ssDNA complexes. The supernatant was transferred to a new tube, extracted with 500 µL of phenol, and centrifuged again at 14,000 rpm for 2 min. The recovered supernatant was supplemented with 5 µL of glycogen (4 µg/µL, total 20 µg), followed by the addition of 1,000 µL ethanol and centrifugation at 14,000 rpm for 15 min. The pellet was washed with 1,000 µl ethanol, centrifuged at 14,000 rpm for 10 min, and then dissolved in 100 µL EcoRI buffer at 37°C for 15 min before heat inactivation at 80°C for 20 min. The resulting cccDNA samples were used for qPCR.

### RT-qPCR detection of total HBV RNA in cytoplasmic samples

Total HBV RNA levels were determined in each of 80 rcDNA samples prepared from untreated mice 842 and 836 (representing the amplification phase, *N* = 40), 831 and 987 (representing the maintenance phase, *N* = 40). Specifically, 20 cccDNA and 20 rcDNA samples from each liver were tested. Average RNA concentrations were approximately 0.5 µg/µL in rcDNA samples. A260/280 ratios varied narrowly between 1.98 and 2.08. All RNA samples were 10-fold diluted and then 2 µL were used for RT-qPCR with TaqMan Fast Virus 1-Step Master Mix (Thermo Fisher 4444432) and primers/probe located in the S gene (rcDNA for qPCR in Table 3). rcDNA was also detected in the same rcDNA samples without RT in the same plates with the same primers/probe for RNA detection. The detected HBV RNA levels were approximately 10 times higher than those of rcDNA in the same samples. The net RNA copies are plotted after subtracting rcDNA copies in the same samples. The ratios of RNA copies/cell to cccDNA copies/cell were calculated using the total net RNA copies (nuclear RNA copies + cytoplasmic RNA copies).

### RT-qPCR detection of human Ki67 RNA levels in 380 cytoplasmic samples

One set of pre-stocked human Ki67 RNA primers/probe system (FAM-MGB, Hs01032435_g1) was purchased from ThermoFisher Scientific. This detection system generates a 179 bp-long amplicon that was isolated for the preparation of qPCR standards. Two microliter from each cytoplasmic rcDNA sample containing cellular RNAs was used for RT-qPCR detection of human Ki67 RNA with the same TaqMan Fast Virus 1-Step Master Mix (Thermo Fisher 4444432). Correlation analysis between Ki67 RNA and cccDNA in the same samples was conducted using scatter plots, generating correlation trendlines and R^2^ values.

The rcDNA samples were consistently stored at −20°C, and all handling procedures were conducted in a biosafety cabinet with an air blower on. All tips, plates, tubes, and solutions used were nuclease-free. The Ki67 RNA levels in 20 rcDNA samples isolated from mouse 805 were analyzed on two occasions, separated by approximately 16 months. The results from two tests are comparable (0.23 vs 0.33 copies/cell), suggesting no noticeable RNA degradation during the 16-month storage.

### qPCR of serum HBV DNA and intrahepatic rcDNA and cccDNA

Primers and probe sequences for the detection of serum HBV DNA and intracellular rcDNA by qPCR are listed in Table 5, while primer sequences for the detection of cccDNA flank the gap region, and the probe is placed immediately after the DR1 sequence (Table 6). The specificity of the listed cccDNA primers and probe can discriminate against rcDNA amplification by 300-fold to 6,000-fold. qPCR was performed using TaqMan fast advanced master mix (ThermoFisher cat no:4444558) in a QuantStudio 3 instrument (ThermoFisher cat no: A28136) that accommodates 0.1 mL 96-well hard-shell plate.

**TABLE 5:** Evaluation of the rcDNA signal in uninfected nuclei with or without mixing mouse 987 liver lysate

	Liver ID: B20		Liver ID: B46	
	Alone	Mixed with 987 lysate	Alone	Mixed with 987 lysate
Number of duplexing dPCR	96	96	80	96
Number of positive rcDNA signal (≥500)	0	0	2	2

**TABLE 6:** Positions and sequences of cccDNA and rcDNA primers and probes

Target	Primer ID	Nucleotide position	Sequence
rcDNA in qPCR	Pan122F	520–537	ccagcacgggaccatgc
	Pan160R	655–637	tgaggcccactcccatagg
	S probe	563–583	FAM′ tgttgctgtacaaaaccttcg
rcDNA in dPCR	rcDNA forward	1345–1370	gtcctctctcggaaatacacctcctt
	rcDNA reverse	1454–1438	tccgcgggattcagcgc
	rcDNA probe	1372–1405	Cy5′ ccatggctgctcgggtgtgctgccaactggatcc
cccDNA in qPCR and dPCR	HBVcccF	1550–1570	cgtctgtgccttctcatctgc
	HBVcccR	1885–1868	aaggcacagcttggaggc
	cccDNA probe	1835–1862	FAM′ ctaatcatctcttgtacatgtcccactg

All standards used for qPCR were calibrated with the Absolute Q digital PCR.

### Absolute Q (ABQ) digital PCR of cccDNA

Absolute Q (ABQ) digital PCR of cccDNA was performed to validate cccDNA copies/cell initially computed by qPCR, using the ThermoFisher Absolute Q Digital PCR system (cat. no. A52864). Briefly, a 9.1 µL reaction mix was prepared consisting of 1.8 µL of 5 × DNA dPCR mix (ThermoFisher cat. no. A52490), 0.5 µL of 20 × primers/probe mix (final concentrations: 900 nM each primer and 250 nM probe), 1 µL of cccDNA sample, and 5.8 µL of DNase- and RNase-free water. A 9 µL aliquot of the reaction mix was then loaded into a single well of a microfluidic array plate (MAP; ThermoFisher cat. no. A53301). Digital PCR was carried out with the following cycling conditions: 10 min preheating at 96°C, followed by 40 cycles of 5 s at 96°C and 15 s at 60°C. Data reports were generated using QuantStudio Absolute Q Digital PCR software.

The sensitivity of dPCR is a single copy per microchamber, and the result is deemed valid if the Rox fluorescent signal was read in >19,000 of 20,480 microchambers for each sample.

### Procedures and principles of simultaneous detection of cccDNA and rcDNA by ABQ duplexing digital PCR in the same nuclei

For detection of both cccDNA and rcDNA in the same nuclei, 20–30 mg of liver tissue was homogenized in 500 µL of homogenization buffer (10 mM Tris-HCl (pH 7.5), 3 mM MgCl₂, 0.25 M sucrose, and 0.05% Triton X-100), and nuclei were pelleted by centrifugation and resuspended in homogenization buffer containing 2 µg/mL ethidium bromide. Individual nuclei were then singly sorted and deposited into wells of 96-well plates, digested with proteinase K (0.5 mg/mL, 60 min), and heat-inactivated at 80°C for 15 min. The released HBV DNA was subsequently linearized by NcoI digestion and subjected to ABQ duplexing dPCR for simultaneous detection of cccDNA and rcDNA.

### Principles of simultaneous detection of cccDNA and rcDNA by ABQ duplexing digital PCR (ABQ dPCR) in the same nuclei

The ABQ dPCR instrument can simultaneously detect four fluorescent signals of FAM, VIC, ABY, and JUN/Cy5, which allows detecting four different targets in the same reaction (Multiplexing). The emission wavelengths of FAM and Cy5 are 517 and 670 nm, respectively, and there is no overlap in their wavelength spectrum. Thus, FAM was selected for labeling the cccDNA probe and Cy5 for labeling the rcDNA probe to detect both molecules in the same reaction, called duplexing dPCR.

The specificity of cccDNA detection is provided through cccDNA-specific primers that flank the gap region in the HBV genome and the cccDNA-specific probe that is placed immediately after the DR I sequence (Table 6). Figure 5E shows an example of its specificity for cccDNA and rcDNA detection.

### Linearized cccDNA template cannot generate a fluorescent signal with the rcDNA primers/probe detection system

HBV DNA released from each of the deposited nuclei was subjected to NcoI digestion to exclude cccDNA from detection with rcDNA primers/probe. Plus strand in rcDNA is only partially synthesized, containing a single-stranded gap of 600–2,100 nucleotides at 3′ end (60). Since NcoI is located between nt1372 and 1376, close to the 3′ end of the plus strand. Therefore, it is most likely present in a single-strand sequence in rcDNA molecules (60). Thus, NcoI will linearize cccDNA but cannot cut rcDNA (Fig. 5A and B).

The NcoI linearized cccDNA sequence starts with C at nt1373 (5′) and ends with C at nt1372 (3’) (Fig. 5C). The rcDNA forward primer will bind the 3′ end of the linearized cccDNA, but the rcDNA probe binds its 5′ end. The Taq DNA polymerase that binds the forward primer at the 3′ end cannot reach the probe that is located at the 5′ end, thus cannot cut off the 1st base C with Cy5 dye through its 5′−3′ exonuclease activity, and the Cy5 fluorescent signal cannot be generated (Fig. 5D). Cy5 fluorescent signal will be generated if both rcDNA forward primer and probe bind a continuous template comprising nt1345 to nt1454 sequentially, that is, F primer binds upstream of the probe binding position (Fig. 5C), which only occurs in rcDNA after NcoI cut. Thus, rcDNA, but not cccDNA, will be specifically detected with rcDNA primers/probe following NcoI cut.

We used an HBV DNA plasmid (an ADW subtype monomer cloned into the Psp65 vector) that was linearized by NcoI as a surrogate cccDNA molecule to validate that cccDNA was not detected by the rcDNA probe/primers. When only the cccDNA sample (approximately 8–10 copies/μL) was used for the test, 8 copies of cccDNA molecules were only detected by the FAM-labeled cccDNA probe; however, no positive signal was detected by the Cy5-labeled rcDNA probe in duplexing dPCR (Fig. 5E1A and B). When only rcDNA (approximately 30 copies of rcDNA/μL, extracted from an infected liver) was included for test, 27 copies of rcDNA were detected only by Cy5-labeled rcDNA probe but not by FAM-labeled cccDNA probe in duplexing dPCR (Fig. 5E2A and B). When the above cccDNA and rcDNA samples were mixed for test, 8 copies of cccDNA and 30 copies of rcDNA were detected by FAM-labeled cccDNA probe and Cy5-labeled rcDNA probe, respectively, in duplexing dPCR (Fig. 5E3A and B).

### Detected rcDNA molecules in the nuclei were not non-specifically bound to nuclei

To evaluate the possibility that the detected rcDNA in sorted individual nuclei was non-specifically bound to the nuclear membrane during the preparation of nuclei suspension through homogenization that released virions and capsids into the lysate, we prepared nuclei suspensions from two livers of two uninfected chimeric mice (animal ID HKB-043-020 or B20 and HKB-043-046 or B46 purchased from PheonixBio). Each nuclei suspension was divided into two vials; one was directly used for sorting, and the other was mixed with lysate (containing 0.05% Triton X-100) from mouse 987, who was an untreated control with average 870 copies of rcDNA/cell for 20 minutes, then removed the lysate and dissolved in isotonic buffer (154:1 TE with 0.05% Triton X-100) for sorting. The sorted nuclei from four vials were subjected to duplexing ABQ dPCR. Table 4 shows no significant differences in detecting nuclei with Cy5 intensity ≥500 between two nuclei suspensions mixed with mouse 987 lysate and the two nuclei suspensions without mixing, suggesting that detected rcDNA molecules in sorted nuclei likely derived from released virions and capsids that non-specifically bound nuclei, which is consistent with the concept that HBV capsid mainly utilizes cellular transport machineries, but not diffusion or passive trapping to reach nuclear membrane where interaction between nuclear localization signal on capsid and nuclear import receptors occurs (61, 62) (Table 5).

### Isolating individual HBsAg-positive cells via FACS

Individual hepatocytes were isolated from each liver of mice 516 and 518 on day 218 pi using a two-step collagenase perfusion method (63). Each liver was perfused at 37°C for 10 min at 1.5 mL/min with Ca^2+^-free and Mg^2+^-free Hanks’ balanced salt solution (CMF-HBSS) containing 200 mg/mL ethylene glycol tetraacetic acid (EGTA), 1 mg/mL glucose, 10 mM N-2-hydroxyethylpiperazine-N′-2-ethane sulfonic acid (HEPES). The perfusion solution was then changed to CMF-HBSS containing 0.05% collagenase (Gibco 17101-015), 0.6 mg/mL CaCl2, 10 mM HEPES, and continued for 17-23 min at 1.5 mL/min. The liver was dissected and transferred to a dish; liver cells will be gently disaggregated in the dish with CMF-HBSS containing 10% bovine Alb, 10 mM HEPES. The disassociated cells were centrifuged three times (50 × *g*, 5 min). The pellet was suspended in PBS with 0.05% Triton X-100.

Cell concentration in the suspension was adjusted to 5 × 10^6^/mL, was first stained with 1/100 diluted rabbit anti-HBs antibody (LSBio LS-C683282), then with 1/500 diluted goat anti-rabbit IgG conjugated with FITC (LSBio LS-C60878). After washing, the stained cells were suspended in PBS with 0.05% Triton X-100 for sorting.

Before sorting, a FACS analysis using SSC-A and FSC-A was performed with the Aurora CS Sorter located at the Flow Cytometry Shared Service, University of Maryland, School of Medicine in Baltimore, MD. The gating strategy included gating cells first based on SSC-A values, then using FITC signals to gate HBsAg-positive cells for sorting. Individual HBsAg-positive cells were singly sorted into wells of 96-well plates. The sorted cells were subjected to the same treatment as described for the sorted nuclei before duplexing dPCR detection.

### Immunohistochemical staining of HBsAg and HBcAg on sections

Briefly, formalin-fixed paraffin-embedded liver sections were cut at a thickness of 5 µM and used for HBsAg staining after deparaffinization, proteinase K digestion, and inactivation of endogenous peroxidase with 3% hydrogen peroxide. Rabbit anti-HBs antibody (LS-C683282, LSBio) or rabbit anti-HBcAg antibody (LS-C170914) and goat anti-rabbit IgG conjugated with HRP (LS-C316062, LSBio) were used as primary and secondary antibodies, respectively. DAB chromogen kit (ACH500-IFU, CP Lab Chemicals) was used for color development.

### Calculation of HBsAg copies/cell

To calculate copy numbers of HBsAg per cell, cellular HBsAg in liver lysates was determined with qHBsAg ELISA, resulting in IU per 500 µL lysate, which was converted to ng/500 µL, then to copies/cell.

Major parameters for the calculation include the following:

- 1 IU of HBsAg ADR subtype is approximately equal to 1 ng (HBsAg ELISA manual, Bio-rad).
- 1 ng of HBsAg equals 2.74e8 copies using 2.16 MDa molecular weight of a HBsAg particle (64)
- 1 mg of liver tissue contains approximately 1.39e5 cells (65)

### Statistical analysis

HBsAg (IU/mL), HBV DNA (copies/mL), and antibody levels (µg/mL) are expressed as mean ± standard deviation (SD). Average intracellular rcDNA and cccDNA levels are expressed as copies/cell. The number of cells per sampling was calculated using sample weight (mg) multiplied by 1.39E5 cells per mg liver tissue (65), then normalized by a factor of 0.7 by considering 70% of liver cells are human hepatocytes based on the Replacement Index (76%–93%) among the chimeric mice provided by PheonixBio. The formula to calculate copies/cell is listed below:


$$Copies/cell=\frac{Total\;rcDNA\;or\;cccDNA\;copies\;in\;a\;liver\;sample}{Sample\;weight\;\left(mg\right)\times 1.39e5\;\times 0.7}$$

## Data Availability

All data supporting the findings of this study are available from the corresponding author upon reasonable request.
